# Repeated Radionuclide therapy in metastatic paraganglioma leading to the highest reported cumulative activity of ^131^I-MIBG

**DOI:** 10.1186/1748-717X-7-8

**Published:** 2012-01-25

**Authors:** Samer Ezziddin, Amir Sabet, Yon-Dschun Ko, Sunny Xun, Alexander Matthies, Hans-Jürgen Biersack

**Affiliations:** 1 Department of Nuclear Medicine, University Hospital, Bonn, Germany; 2 Internal Medicine, Johanniter Hospital, Bonn, Germany

**Keywords:** Cumulative activity, I-131-MIBG, Neuroendocrine tumors, Radionuclide therapy, Metastatic paraganglioma

## Abstract

^131^I-MIBG therapy for neuroendocrine tumours may be dose limited. The common range of applied cumulative activities is 10-40 GBq. We report the uneventful cumulative administration of 111 GBq (= 3 Ci) ^131^I-MIBG in a patient with metastatic paraganglioma. Ten courses of ^131^I-MIBG therapy were given within six years, accomplishing symptomatic, hormonal and tumour responses with no serious adverse effects. Chemotherapy with cisplatin/vinblastine/dacarbazine was the final treatment modality with temporary control of disease, but eventually the patient died of progression. The observed cumulative activity of ^131^I-MIBG represents the highest value reported to our knowledge, and even though 12.6 GBq of ^90^Y-DOTATOC were added intermediately, no associated relevant bone marrow, hepatic or other toxicity were observed. In an individual attempt to palliate metastatic disease high cumulative activity alone should not preclude the patient from repeat treatment.

## Background

Targeted radiotherapy with ^131^I-metaiodobenzylguanidine (^131^I-MIBG) provides a generally well tolerated treatment in chromaffin tumors; i.e. neuroblastoma, pheochromocytoma, and paraganglioma [1-4]. Hematotoxicity is the primary side effect depending on the administered activity, metastatic bone marrow infiltration and other performed treatment modalities as chemotherapy [5-7]. Treatment protocols mostly use activities between 3.7 and 11.1 GBq (100-300 mCi) per course [8,9]. Newer schedules use 14.8-18.5 GBq (400-500 mCi) per treatment course and high-dose concepts may incorporate an initial dose of > 22,2 GBq (600 mCi) supported by potential autologous stem-cell rescue (ASCR) [10,11].

However, there is no existing recommendation for repeat treatment or maximum cumulative activity. While the common total administered activity per patient varies between 10 and 40 GBq [9,12] occasional reports of higher values up to 70 GBq do exist [10,13-16]. One patient with metastatic pheochromocytoma was noted to receive as much as 85.9 GBq (2321 mCi) ^131^I-MIBG [17].

We report on a case of metastatic paraganglioma undergoing repetitive MIBG treatment during the course of disease, resulting in an extraordinary high cumulative activity of 3 Ci without any observed dose limiting toxicity.

## Case presentation

**Figure 1:**
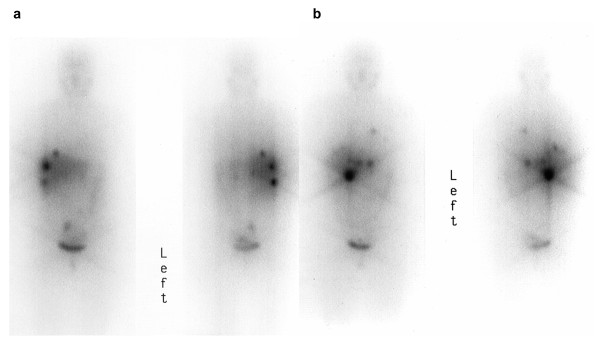
**Post-therapeutic ^131^I-MIBG scans (anterior and posterior view) of the 1^st ^(**a**) and 5^th ^(**b**) therapy showing the well-accumulating metastases in the early to mid stages of the disease**.

A 52-year-old female with a highly functional retroperitoneal paraganglioma and previous tumorectomy was referred for ^131^I-MIBG therapy in recurrent hepatic metastatic disease. The patient suffered from hypertension, tachycardia and sweating. ^131^I-MIBG therapy was initiated after confirmation of tumor uptake in a diagnostic MIBG scan. The treatment was performed using 11.1 GBq (300 mCi) ^131^I-MIBG of high specific activity (GE Healthcare, Amersham, Germany), given intravenously by slow infusion. The minimum interval between two courses of ^131^I-MIBG was eight weeks. Drugs known to interfere with MIBG uptake were discontinued before admission to treatment [18]. Thyroid uptake of free radioiodine was blocked either by sodium perchlorate, 600 mg p.o. three times a day, administered for 3-6 weeks (treatment cycle no.1-6) or by potassium iodide 100-200 mg per day plus levothyroxine 100 μg/d for 2-4 weeks (treatment cycle no. 7-10). Blood pressure and heart rate were monitored during ^131^I-MIBG infusion. Posttherapeutic ^131^I-MIBG imaging was performed four to six and eight to ten days after therapy, with whole body and planar scans using a dual head large-field-of-view gamma camera (Picker Prism 2000 XP, Philips Medical Technology, USA) with a high-energy collimator. Toxicity was evaluated by extensive laboratory tests including complete blood count and liver function tests. Treatment response was assessed by CT and MRI, posttherapeutic MIBG scintigraphy, 24-h urine sampling and monitoring of catecholamine-related symptoms. For radiographic tumour response the Response Evaluation Criteria of Solid Tumors (RECIST) criteria were applied. The MIBG scintigraphy was added for evaluation of bone metastases (classically non-measurable disease in RECIST), i.e. MIBG-positive bone metastases becoming MIBG-negative with accompanying sclerotic changes in CT were interpreted as non-vital. Biochemical response was classified as CR (complete response) in complete normalization and partial response (PR) in more than 50% reduction of catecholamine levels. Symptomatic response was categorized into complete (CR) and partial (PR) resolution of functional symptoms. The first four MIBG treatment courses-given within seven months-produced a gradual and increasing symptomatic, biochemical and radiographic tumour response corresponding with a partial remission (PR). Figure 1 shows the first posttherapeutic scan (a). Partial hepatectomy was performed and left the patient with no detectable residual tumor. The complete radiological and functional remission lasted almost two years, when the patient experienced a relapse with appearance of a few liver and lung metastases as well as a retroperitoneal tumour lesion (Figure 1, b).

**Figure 2:**
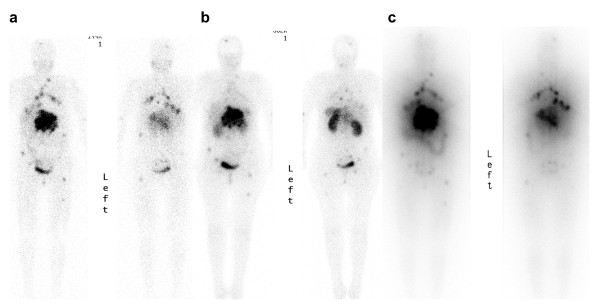
^**123**^**I-MIBG (**a**) and ^111^In-pentetreotide (**b**) scans before MIBG therapy no. 9, followed by the post-therapeutic ^131^I-MIBG scan (**c**): Later stage metastases to the lungs, liver, lymph nodes, soft tissue and bone, still displaying avid tracer uptake. The ^111^In-pentetreotide scan shows no additional lesions compared to the MIBG scans**.

Four more courses of ^131^I-MIBG (cycle no. 5-8) were subsequently given within one year. Following the sixth ^131^I-MIBG cycle the sodium perchlorate used for thyroid blockade caused the patient fever, chills and a rash, and oral iodine (100 mg per day) was used instead thereafter. Again, a partial remission was achieved, but progression occurred less than one year later with development of a clinically predominant large metastatic liver lesion. Since this centrally located lesion proved unresectable during surgery, selective portal vein ligation of the affected liver segments was performed. This procedure was followed by two courses of Y-90-DOTATOC (performed at the University Hospital Basel, Switzerland, with a total dose of 12.6 GBq Y-90) after confirmation of tumour uptake in somatostatin receptor scintigraphy using ^111^In-pentetreotide. The initial response was good (PR), but shortly later the patient experienced extensive multi-site disease progression. The results of the reassessment at that time with pre- and posttherapeutic scintigraphic imaging are depicted in Figure 2, showing widespread metastases. Since the concomitant scintigraphic somatostatin receptor evaluation showed no additional tumour lesions compared to the MIBG scan, there was no indication of dedifferentiation (loss of MIBG-accumulating) of tumour cells. At this advanced stage, only disease stabilization and symptomatic relief were achieved by another 2 MIBG treatment cycles, with tumour progression occurring after six months.

**Figure 3:**
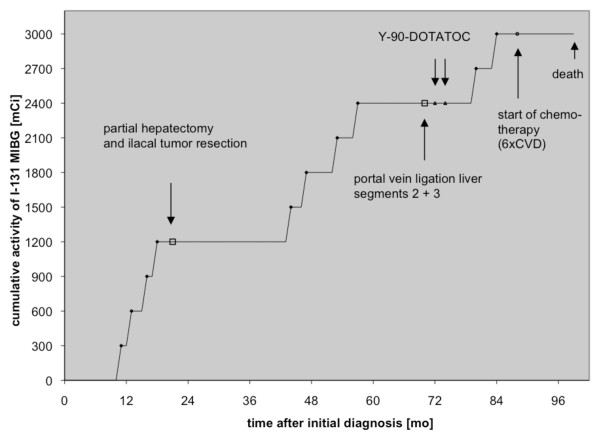
^**131**^**I-MIBG cycles with the resulting cumulative activity and other treatments over the course of time**.

Then, the patient underwent chemotherapy with cyclophosphamide, vincristine and dacarbazine (CVD regimen) with temporary control of disease, but eventually died of progression. No dose limiting bone marrow toxicity was observed throughout and following chemotherapy. It should be mentioned that stem cell support was not available in this patient. Figure 3 gives an overview of the various treatment applications over the course of time including ^131^I-MIBG with the resulting cumulative activity. The treatment response and hematologic toxicity of all ^131^I-MIBG courses is given in Table 1. The only observed adverse effect was a mild to moderate bone marrow toxicity seen after some of the earlier courses of MIBG therapy. It might have been caused by the perchlorate medication used for thyroid blockade at therapy no. 1-6, as this agent is known to cause reversible leukopenia in higher doses as been used in our case [19]. It is noteworthy that no serious toxicity was observed throughout the entire life-span although the high administered dose met potential predisposing factors for liver failure such as previous partial hepatectomy, selective portal vein ligation and liver metastases.

**Table 1:** Treatment response and toxicity.

Tumour lesions	Course of ^131^I-MIBG (no.)	Toxicity leukopenia (grade^a^)	Response			
			**Sympt**.	**Bioch**.	**Radiol**.	TTP^b^
three liver mets, one Ln met	1^st^	0	PR	PR	PR	33
	2^nd^	0	PR	PR	PR	33
	3^rd^	2	PR	PR	PR	33
	4^th^	1	PR	PR	PR	33

no tumour (CR) after surgery						

retroperit. tumour mass, liver and lung mets (< five each)	5^th^	0	CR	PR	PR	23
	6^th^	1	CR	PR	PR	23
	7^th^	1	CR	PR	PR	23
	8^th^	0	CR	PR	PR	23

PR in number and size						

multiple mets to the lungs, liver, bone, soft tissue, Ln	9^th^	0	PR	SD	SD	6
	10^th^	0	PR	SD	SD	6

a: common toxicity criteria (CTC); b: time to progression in months; CR: complete response; PR: partial response; SD: stable disease

## Conclusions

We report the uncomplicated administration of 111 GBq (= 3 Ci) cumulative activity of ^131^I-MIBG without serious toxicity. This case underscores the therapeutic potential of ^131^I-MIBG therapy in metastatic disease as well as inter-individual dose-tolerance variability. This, to our knowledge, represents the highest reported value and compares to the outstanding cumulative activity of 85.9 GBq in one patient noted in a larger series before [17]. In an individual attempt to palliate metastatic disease high cumulative activity alone should not preclude the patient from repeat treatment.

### Consent

Written informed consent for publication of this Case report and any accompanying images was obtained from the patient's relative. A copy of the written consent is available for review by the Editor-in-Chief of this journal.

## Competing interests

The authors declare that they have no competing interests.

## Authors' contributions

Conception and idea of the work: S.E., HJ.B.; Collection and assembly of data or imaging: S.X., A.M., S.E., YD.K.; Drafting of the article: S.E., A.S., HJ.B.; Critical article revision for important intellectual content: HJ.B., YD.K.. All authors read and approved the final manuscript.
